# Neurologic Manifestations of the World Health Organization's List of Pandemic and Epidemic Diseases

**DOI:** 10.3389/fneur.2021.634827

**Published:** 2021-02-22

**Authors:** Caleb R. S. McEntire, Kun-Wei Song, Robert P. McInnis, John Y. Rhee, Michael Young, Erika Williams, Leah L. Wibecan, Neal Nolan, Amanda M. Nagy, Jeffrey Gluckstein, Shibani S. Mukerji, Farrah J. Mateen

**Affiliations:** ^1^Massachusetts General Hospital (MGH)-Brigham Neurology Residency Program, Boston, MA, United States; ^2^Massachusetts General Hospital (MGH)-Brigham Pediatric Neurology Residency Program, Boston, MA, United States; ^3^Department of Neurology, Massachusetts General Hospital, Boston, MA, United States

**Keywords:** epidemic, pandemic, neuroviral epidemics, clinical neurology, tropical medicine

## Abstract

The World Health Organization (WHO) monitors the spread of diseases globally and maintains a list of diseases with epidemic or pandemic potential. Currently listed diseases include Chikungunya, cholera, Crimean-Congo hemorrhagic fever, Ebola virus disease, Hendra virus infection, influenza, Lassa fever, Marburg virus disease, *Neisseria meningitis*, MERS-CoV, monkeypox, Nipah virus infection, novel coronavirus (COVID-19), plague, Rift Valley fever, SARS, smallpox, tularemia, yellow fever, and Zika virus disease. The associated pathogens are increasingly important on the global stage. The majority of these diseases have neurological manifestations. Those with less frequent neurological manifestations may also have important consequences. This is highlighted now in particular through the ongoing COVID-19 pandemic and reinforces that pathogens with the potential to spread rapidly and widely, in spite of concerted global efforts, may affect the nervous system. We searched the scientific literature, dating from 1934 to August 2020, to compile data on the cause, epidemiology, clinical presentation, neuroimaging features, and treatment of each of the diseases of epidemic or pandemic potential as viewed through a neurologist's lens. We included articles with an abstract or full text in English in this topical and scoping review. Diseases with epidemic and pandemic potential can be spread directly from human to human, animal to human, via mosquitoes or other insects, or via environmental contamination. Manifestations include central neurologic conditions (meningitis, encephalitis, intraparenchymal hemorrhage, seizures), peripheral and cranial nerve syndromes (sensory neuropathy, sensorineural hearing loss, ophthalmoplegia), post-infectious syndromes (acute inflammatory polyneuropathy), and congenital syndromes (fetal microcephaly), among others. Some diseases have not been well-characterized from a neurological standpoint, but all have at least scattered case reports of neurological features. Some of the diseases have curative treatments available while in other cases, supportive care remains the only management option. Regardless of the pathogen, prompt, and aggressive measures to control the spread of these agents are the most important factors in lowering the overall morbidity and mortality they can cause.

## Introduction

The World Health Organization (WHO) provides guidance on diseases that are international threats to populations with a focus on diseases of epidemic and pandemic potential (1). Close observation of these infectious diseases is required to ensure prevention, foster early detection, mitigate further spread, and provide supportive care to those affected. To date, of all of the millions of pathogens globally, only ~20 infections are listed as having epidemic or pandemic potential by the WHO. While some may be familiar to the neurologist, others may be only known to practitioners in tropical settings or locations where few neurologists practice. Here we synthesize the available knowledge on the neurological manifestations of the WHO's listed diseases of epidemic and pandemic potential by pathogen.

## Materials and Methods

Searches were performed using the keywords of the disease and infectious organism (if different) as well as the terms “neurology,” “brain,” “nervous system,” and related terms for each of the listed disease of epidemic or pandemic potential on the WHO website as of April 1, 2020. Search engines included PubMed, Google Scholar, and the reference lists of articles found through those searches. All searches took place in spring 2020 with updates continuing as more information became available, particularly on evolving pandemics, such as SARS-CoV-2, influenza, Ebola Virus disease, and *Neisseria meningitidis* meningitis until September 2020. Articles in English or with an abstract in English were included, using data sources dating from 1934 until present.

### Data Availability Statement

Data sharing is not applicable to this article as no new data were created or analyzed in this study.

## Results

### Epidemic and Pandemic Diseases

#### Chikungunya

Chikungunya virus is a positive-sense alphavirus most often spread by *Aedes* mosquitos as a direct infection to humans, although it can rarely pass through maternal-fetal transmission or blood products. Chikungunya presents with a systemic course that is relatively uniform across individuals, but neurologic manifestations can be more heterogeneous. Its systemic course begins with an incubation period from 1 to 12 days and then progresses to fever, malaise, and headache, as well as a characteristic symmetric arthralgia in as many as 90% of patients (2). Chikungunya means “that which bends up” in Kimakonde, the language of the ethnic group in whom the disease was first identified (3), referring to the stooped posture individuals with the disease adopt due to joint pain (Tables 1, 2).

**Table 1:** Overview of presentation, diagnosis, and management of the WHO Diseases of Epidemic & Pandemic Potential.

**Disease name**	**Vaccine available**	**Highest level of evidence for neurological manifestations**	**Affects CNS, PNS, or both**	**Most common systemic presentation**	**Most common neurological presentation**	**Pathogen found in CSF**	**Neuroimaging findings**	**Management**
Chikungunya	No	Case series, case-control study, cross-sectional study (with systematic review covering all of these)	Majority CNS, 25% PNS	Fever, malaise, arthralgias	Encephalopathy, myelitis	IgM found in CSF	Punctate foci of T2 signal in periventricular, limbic areas	Supportive care
Cholera	Yes	Retrospective analysis, case series	CNS	Diarrhea, dehydration	Meningitis, abscess	Yes	Uncertain	Aggressive volume repletion plus antibiotic therapy
COVID-19	No	Case series, case reports	CNS, PNS	Fever, chills cough, shortness of breath, fatigue, myalgias, headache, loss of taste or smell, nausea, vomiting, diarrhea	Encephalitis, headache, Guillain-Barre syndrome, myelitis	Uncertain	Uncertain	Supportive care
Crimean-Congo hemorrhagic fever	No	Prospective cohort study, case series	CNS	Fever, myalgias, GI disturbance, hemorrhage	Headache, dizziness, encephalopathy, ICH	Uncertain, viral RNA found in brain tissue in animal models	Normal findings, or diffuse hemorrhage	Supportive care
Ebola virus disease	Yes	Large prospective cohort study	Majority CNS	High fevers, fatigue, myalgias, conjunctival injection, chest pain, arthralgias.	Headache, diffuse weakness, encephalopathy	Yes	Punctate T2 and DWI lesions in corpus callosum and cerebral white matter^*^	Supportive care
Hendra virus infection	No human vaccine available	Case series	CNS	Fever, respiratory symptoms	Headache, meningitis, encephalitis, seizures	Antibodies found in CSF; likely viral inclusion bodies on autopsy	Early isolated cortical signal, later deep white matter involvement	Supportive care
Influenza	Yes	Large prospective cohort study	CNS and PNS	Fever, headache, myalgia, malaise	Headache, seizure, encephalopathy	Rarely	Increased DWI signal in cortex and adjacent white matter	Supportive care
Lassa fever	No	Retrospective case series	CNS	80% asymptomatic; otherwise malaise, fever, bleeding	Sensorineural hearing loss, encephalopathy	Yes	Uncertain	Supportive care
Marburg virus disease	No	Case series, case studies	CNS	High fever, headache, malaise, myalgias, diarrhea, hemorrhage	Encephalitis, headache, hemorrhage	Antibodies in CSF	Uncertain	Supportive care
MERS-CoV	No	Retrospective case series	CNS and PNS	Respiratory disease with fever, cough, shortness of breath	Headache, encephalopathy, seizures, focal motor deficits, weakness, paresthesias, ophthalmoplegia and ataxia	No	Patchy diffusion restriction and T2 hyperintensity	Supportive care
Monkeypox	No, although some cross-reactivity from smallpox vaccine	Two case reports	CNS	Asymptomatic, or fevers and rash	Encephalitis	Uncertain	Uncertain	Supportive care
Neisseria meningitidis meningitis	Yes	Large retrospective cohorts and reviews	CNS	Fever, nausea, vomiting, myalgias, petechial rash, sepsis	Headache, neck stiffness, altered mental status	Yes	Non-specific T2 abnormalities	Antibiotic therapy plus prophylaxis for close contacts
Nipah virus infection	No	Case series	CNS	Viral pneumonia, fever	Fulminant encephalitis with headache and meningismus	Antibodies in CSF	Many asymmetric, small lesions in subcortical white matter	Supportive care
Plague	Yes, although no longer available in U.S.	Retrospective analysis, case series	CNS	Buboes, high fever, shock	Meningitis	Yes	Uncertain	Antibiotic therapy plus prophylaxis for face-to-face exposures
Rift valley fever	No, but an inactivated vaccine has been trialed in humans	Prospective study, Case-control study, cross-sectional study	CNS	Mild flu-like illness, fever, joint paint pain, muscle ache	Encephalopathy, meningoencephalitis	No, but found in animal studies	Bilateral asymmetrical cortical hyperintense areas^*^	Supportive care
SARS	No	Case studies, case reports	CNS	Fever, cough, shortness of breath, diarrhea	Headache, encephalitis	Yes	Uncertain	Supportive care
Smallpox	Yes, but not commonly available	Case-series, retrospective analysis (for vaccine related complications)	CNS	Fevers, malaise, vesicular/pustular rash	Encephalopathy, headaches	Uncertain	Uncertain	Supportive care; tecovirimat FDA-approved but sparsely tested
Tularemia	No	Case reports	CNS and PNS	Fever, regional tender lymphadenopathy, conjunctivitis, pharyngitis, pneumonia	Overall rare, though meningitis, ataxia, polycraniitis, and Guillain Barre syndrome have been reported	Yes	Meningeal enhancement, scattered T2-intense lesions. Can present with abscess.	Antibiotic therapy
Yellow fever	Yes	Case-series, retrospective study	CNS	Fevers, minority with hemorrhagic fever	Encephalopathy, seizures	Yes	Petechiae, perivascular hemorrhages and edema	Supportive care
Zika virus disease	No	Large retrospective cohorts	CNS and PNS	Fever, arthralgias, headache, rash	Guillain-Barre syndrome, fetal microcephaly	Yes	Typical GBS findings; infants: subcortical calcifications and microcephaly	Supportive care

* *Characterized only in individual case report*.

**Table 2:** Overview of epidemiology of the WHO Diseases of Epidemic & Pandemic Potential.

**Disease name**	**Organism type**	**Organism name**	**Animal Reservoir (yes, specify) e.g., horses, bats**,	**WHO World Region**	**Reported in children, adults, elderly or all of the above**
Chikungunya	Virus	*Chikungunya virus*; *Togaviridae* family (positive-sense ssRNA)	*Aedes* mosquitos with reservoir in birds and rodents	Primarily Africa and SE Asia, now Europe & N America	All of the above
Cholera	Bacterium	*Vibrio cholerae (GN), specifically non-O1 serogroups*	No	Mostly Africa and Asia	Mostly children, some adults/elderly
COVID-19	Virus	*SARS-CoV-2; Coronaviridae* family (positive-sense ssRNA)	Likely, exact source unclear	All	Mostly adults
Crimean-Congo hemorrhagic fever	Virus	*Crimean-Congo hemorrhagic fever virus*; *Nairoviridae* family (negative-sense ssRNA)	*Hyalloma* tick with reservoir in cattle, goats, sheep, hares, and ostriches	Africa, the Balkans, Middle East, Asia	All of the above
Ebola virus disease	Virus	*Ebola virus*; Filoviridae family (negative-sense ssRNA)	Animals hunted for bushmeat, including bats, are a suspected reservoir	West Africa, Democratic Republic of the Congo	All of the above
Hendra virus infection	Virus	Hendra virus; *Paramyxoviridae* family (negative-sense ssRNA)	*Pteropus* bats, horses	Asia, Pacific	Adults
Influenza	Virus	*Influenza virus*; *Orthomyxoviridae* family (negative-sense ssRNA)	Waterfowl	All continents	All of the above
Lassa fever	Virus	*Lassa virus*; A*renaviridae* family (negative-sense ssRNA)	No	West Africa	Adult
Marburg virus disease	Virus	*Marburgvirus; Filoviridae* family (negative-sense ssRNA)	Yes; African fruit bat	Africa	Mostly adults
MERS-CoV	Virus	Middle East respiratory syndrome-related coronavirus; *Coronaviridae* family (positive-sense ssRNA)	Dromedary camels, bats	Eastern Mediterranean (origin); cases reported in all regions	Adult
Monkeypox	Virus	*Monkeypox virus*; *Poxviridae* family (dsDNA)	Monkeys, rats, squirrels	Africa, U.S.	Children
Neisseria meningitidis meningitis	Bacterium	*Neisseria meningitidis;* GN diplococcus	No	All continents, with epidemic outbreaks in Africa's meningitis belt	All of the above
Nipah virus infection	Virus	*Nipah henipavirus; Paramyxoviridae* family (negative-sense ssRNA)	*Pteropus* bats	Primarily Africa and SE Asia	Children and adults
Plague	Bacterium	*Yersinia pestis* (GN, non-motile)	Tropical rat flea (*Xenopsylla cheopis*) or domestic cats	All continents except Oceania	Majority children, some adults
Rift valley fever	Virus	*Rift valley fever virus; Phenuiviridae* family *(*negative-sense ssRNA)	*Aedes* mosquitoes	Africa, Arabian peninsula	All of the above
SARS	Virus	*SARS-CoV-1; Coronaviridae* family *(ssRNA)*	Horseshoe bat	China	Mostly adults
Smallpox	Virus	*Variola major and minor; Poxvirus* family (dsDNA*)*	No	Eradicated	All of the above
Tularemia	Bacterium	*Francisella tularensis* (GN coccobacllus)	Rabbits, hares, beavers and muskrats	North America, Europe, Asia, and Middle East. Uncommon in Australia and South America.	All of the above, though mostly adults
Yellow fever	Virus	Yellow fever virus; flaviviridae (positive-sense ssRNA)	*Aedes* mosquitoes	African, South America	All of the above
Zika virus disease	Virus	Zika virus; flaviviridae (positive-sense ssRNA)	*Aedes* mosquitoes	Global	All of the above

*ds,double-stranded; ss, single-stranded; GN, Gram-negative*.

The most common clinical presentation of neurologic Chikungunya is encephalopathy, which can be present in up to 40% of individuals with neurologic involvement. Case series of numerous epidemics have also reported meningoencephalitis, Guillain-Barré Syndrome (GBS), myeloradiculitis, facial palsy, sensorineural deafness, optic neuritis, and more (4, 5). Approximately 75% of adult and pediatric cases have a pure central nervous system (CNS) phenotype, while ~13% have a pure peripheral nervous system (PNS) disorder. Approximately 9% have disorders of both the CNS and PNS.

The time course of neurologic signs varies by the syndrome; encephalopathy often appears concurrently with initial systemic symptoms, occasionally lagging by 1 to 2 days. Spinal disease, with weakness and occasional paresthesias of multiple limbs, can appear at symptomatic onset or with a delay of up to 3 weeks. GBS appears weeks after the initial illness, consistent with its usual presentation (5).

The exact proportion of individuals infected with Chikungunya who exhibit neurologic involvement is unclear, but it appears to be the most common “atypical” involvement in severe disease. Case series during epidemics have reported neurologic involvement ranging from 0.1% (5) to 16.3% (6). A neurological disorder was the primary issue in 61% (7) and 79% (8) of more severely affected patients in intensive care units.

Diagnosis of chikungunya is with real-time reverse-transcription polymerase chain reaction (RT-PCR) or enzyme-linked immunosorbent assay (ELISA) of the serum depending on the timing of testing. Within 5 days of initial symptom onset, serum RT-PCR for viral RNA has up to 100% sensitivity and 98% specificity, while ELISA for immunoglobulin M (IgM) is appropriate for patients who have shown clinical symptoms for at least 6–8 days (9, 10). Viral RNA has not been shown in the CSF of affected individuals, although viral particles have been found in glial cells of infected mice (11). Treatment of the disease is supportive care, including rest and fluids, as well as acetaminophen or non-steroidal anti-inflammatory drugs to alleviate arthralgias. Corticosteroids have been used in some published case reports but with no data for their efficacy (5). GBS should be treated as per its standard protocols with intravenous immunoglobulins (IVIg) or plasmapheresis.

#### Cholera

Cholera was classified as a pandemic by the WHO in 2010 in light of the disease causing an estimated 28,800–130,000 deaths per year and affecting 3–5 million individuals globally (12). The causative organism *Vibrio cholerae* is a gram-negative, comma shaped bacterium most commonly found in seawater and known to have a number of serogroups. Specifically, serogroups O1 and O139 have been implicated in the majority of intestinal infections that are most common worldwide (13). Non-O1 serotypes have long been associated with more invasive disease phenotype and extraintestinal involvement (14).

Infants and neonates make up the majority of cholera cases with neurologic sequelae, presenting with *V. cholerae* meningitis, abscess, or both. In some cases there appears to have been a specific causative risk factor, as in one case where an individual had eviscerated fish from a nearby reservoir in the family sink 2 weeks prior to presentation (15), or a case where a neonate developed *V. cholerae* O139 septicemia and lethargy in the context of hospital workers managing an adult with bacteremia of the same organism in a nearby ward (16). In other cases no specific source of infection was found, but environmental factors in a resource-limited setting could be implicated. One 10-day-old boy with *V. cholerae* meningitis had no discrete risk factor identified, but recent studies of water in the rural area of India in which he resided had isolated non-O1 non-0139 *V. cholerae* from 41% of drinking water samples as well as 100% of water, sediment, and plankton samples from three nearby lakes (17).

Interestingly, the infant cases reported of cholera meningitis have presented with lethargy, fever, and refusal to feed, but none have presented with the diarrhea typical of intestinal cholera. Prognosis of these infants appears poor; 2 of 6 cases we have been able to identify in the literature have resulted in death, while 3 of the remaining patients have had lasting neurologic sequelae (18).

Neurological consequences of any cholera serotype have also been described in adults, but often with more concrete risk factors for intracranial infection. For instance, one 58-year-old fisherman developed a left-sided paresis and a Babinski sign and was found to have intracerebral abscess and bacteremia with non-cholera toxin-producing, non-O1, non-O139 *V. cholerae*. While he had no recently identified risk factor for the disease beyond seawater contact, he had craniotomy for a subdural hematoma 5 years before presentation that could have allowed the organism intracranial access (19).

The gold standard for diagnosis of cholera is stool culture for *V. cholerae*, although several rapid antigen-based tests are available depending on the resource availability of the area where testing occurs (20). CSF culture has also successfully grown the organism in most cases (17, 18, 21, 22), although in at least one case report, there was no yield (23). Given the rarity of cholera meningitis no large-scale studies have examined antibiotic use, but it is prudent to treat the disease with antibiotics given its poor prognosis. The available literature indicates that most organisms have been susceptible to third-generation cephalosporins, piperacillin/tazobactam, fluoroquinolones, and tetracyclines (18). Supportive care should also be offered.

#### Crimean-Congo Hemorrhagic Fever

Crimean-Congo Hemorrhagic Fever (CCHF) is a hemorrhagic illness caused by infection with the Crimean-Congo hemorrhagic fever virus (CCHFV), a negative-sense RNA virus of the *Nairoviridae* genus of the *Bunyaviridae* family with a genome comprised of three segments (24). CCHF was first recognized in Crimea in 1944 during an outbreak involving Soviet troops and was subsequently named Crimean Hemorrhagic Fever (25). In 1956, an identical pathogen was identified in the former Belgian Congo (current Democratic Republic of the Congo) and named the “Congo Virus”; the names were later consolidated to CCHFV (25). As the most widespread tickborne virus, CCHFV is endemic throughout areas of Africa, southeastern Europe, Asia, and the Middle East (24). Although typically contained, outbreaks have been increasing and the geographic distribution expanding in recent years due to a number of factors including climate change (26, 27).

Infection with CCHFV can occur directly from a bite from an infected *Hyalomma* tick, through the butchering of livestock that act as amplifying hosts for the virus, or via contact with infected bodily fluids (27). The highest risk for infection occurs in the spring and summer, when tick bites are most likely to occur, and often is limited to an isolated rural case. The clinical presentation of CCHF begins with an incubation period of 1–13 days (25). This is followed by a pre-hemorrhagic phase, characterized by the sudden onset of a non-specific febrile illness with myalgias, diarrhea, nausea, and vomiting (27). The hemorrhagic phase follows in 3 to 6 days and can evolve from petechiae to widespread hemorrhage in severe cases, with mortality ranging from 9 to 50% (24). Survivors typically will enter the convalescent phase 15 to 20 days after symptomatic onset (24).

Common neurologic manifestations in CCHF include headache, seen in 68% of patients (28), dizziness, photophobia, neck pain and stiffness, and encephalopathy. Given the extensive hemorrhage often associated with the disease, intracranial hemorrhage (ICH) is an expected finding. There have been reported cases of intraparenchymal, subarachnoid, and subdural hemorrhages diagnosed on imaging (29–31) and on autopsy (32), in addition to fatal cerebellar tonsillar herniation secondary to cerebral edema with associated frontal intraparenchymal hemorrhage (33), While ICH may be found in more severe and often fatal cases, there are also milder forms of illness that are not associated with intracranial abnormalities in spite of clinical findings that implicate CNS involvement (fever, headache, alteration of consciousness, agitation, and myoclonic jerks) (34). Little is reported on the long-term neurologic sequelae of survivors of CCHF.

Despite the frequent lack of CNS involvement on neuroimaging, animal studies have demonstrated the presence of CCHFV antigen in the brain (primarily glial cells and meninges) for certain strains of CCHFV along with neuropathological evidence of gliosis and meningoencephalitis. Viral RNA has also been isolated from ocular samples in mice. There is evidence of vascular congestion in the CNS, indicating a route of entry for CCHFV through the blood-brain-barrier related to the virus's known tropism for endothelial cells (35). Serum RT-PCR for CCHFV RNA is sensitive and specific for the disease, and several real-time tests exist for a rapid diagnosis (36, 37).

### Ebola Virus Disease

Ebolavirus is a virus in the *Filoviridae* family, whose infamy as one of the most dangerous human pathogens has earned it recognition as a potential bioweapon (38, 39). It is an RNA virus that primarily infects macrophages and endothelial cells using the C1 protein associated with Niemann-Pick disease as a receptor. Interestingly, cells derived from patients with Niemann-Pick disease are resistant to infection (39, 40). Transmission to humans occurs through infected animals, and bats are recognized as one of the most important disease reservoirs. Human to human transmission occurs through bodily fluids, and can even be transmitted from people who died from infection, where the virus can remain viable for many days (39). Ebola recently gained new attention following an epidemic of unprecedented magnitude, which spread through West Africa from 2013 to 2016. The estimated case fatality was 50% (41, 42). The size of this epidemic led to accelerated scientific discoveries about the virus, including the numerous neurologic manifestations of disease, and the possibility of disease relapse from immunologically privileged sites in the body (39).

Symptoms of Ebola virus disease (EVD) typically begin after an incubation period lasting 4 to 10 days. Early disease presents with non-specific symptoms, including high fevers, headaches, fatigue, myalgias, and body aches; there may be associated conjunctival injection, chest pain, and arthralgias. A few days later, infected individuals may develop abdominal pain with nausea, copious vomiting and watery diarrhea, which can lead to severe volume depletion and shock. Hemorrhagic disease is a late complication that affects a minority of infected people, and usually manifests with petechiae, ecchymoses, oozing from sites of venipuncture, hematemesis, or melena (39).

There is better understanding of neurologic complications from EVD in the wake of recent epidemics; however, neurologic evaluation of acutely infected patients poses significant challenges. Strict quarantining and extensive personal protective equipment can limit a comprehensive neurologic exam and has largely prohibited lumbar puncture (LP) for CSF analysis, except in rare cases. Acute neurologic manifestations vary in prevalence from very common, to quite rare. Commonly seen neurologic manifestations include headaches, diffuse weakness, and a varying degree of encephalopathy. Rarely, EVD can be complicated by hemorrhagic or ischemic strokes. Cerebellar signs and myoclonus have also been observed, but are considered rare (43). Seizures can occur, and patients can develop a syndrome concerning for meningitis or encephalitis. Neuropsychiatric signs and symptoms vary widely, with a report of ideomotor slowing, aggression, and disinhibition, which may be related to delirium, but also raise concern for CNS involvement (44). LP was performed on three patients with confirmed EVD in Guinea who exhibited worsening neuropsychiatric symptoms, but no meningeal signs. Although biochemical and cytological analysis could not be performed, quantitative reverse transcription polymerase chain reaction (RT-PCR) was positive for EVD in CSF in each case (44). Separately reported, another patient with EVD developed frank meningeal signs, and then underwent LP, which was notable for sterile CSF, no red or white cells, but positive EVD RT-PCR (45). Importantly, one case report identified a patient who was recovering from EVD, but at 2 weeks developed marked neurologic decline, with LP showing an elevated opening pressure. Interestingly, RT-PCR returned positive in CSF, but was negative in the serum (39).

A proportion of people who survive EVD may develop chronic or delayed onset neurologic complications, and cases have surfaced that demonstrate reactivation of virus within immunologically privileged sites. Survivors of a 2007 EVD outbreak in Uganda exhibited headaches, retro-orbital pain, hearing loss, memory problems, and confusion more commonly than controls, 2 years following infection (39, 46). Hearing loss and generalized fatigue have also been reported (39). A large cohort study called PREVAIL III compared 966 antibody-positive EVD survivors to 2,350 antibody negative close contacts with no history of EVD as a control group over a period of 5 years. EVD survivors were found to have urinary frequency, headache, fatigue, muscle pain, memory loss, and joint pain more frequently than controls. On follow up, survivors were also more likely to have an abnormal neurologic exam (47). Uveitis was also more common among survivors, and prior research has demonstrated the persistence of viral RNA in ocular fluid in these individuals, suggesting a pathologic effect of active viral replication (47, 48). Viral relapse causing meningoencephalitis was observed in a 39-year-old Scottish nurse 9 months after a full recovery from typical EVD. She was found to have multiple cranial neuropathies in addition to radiculopathy. LP was performed, with CSF RT-PCR positive for EV. Other cases have been suggestive of EVD relapse in the CNS (39). Neurologists should be aware of the range of neurologic manifestations, and remarkable capacity for the virus to relapse with neurologic symptoms.

#### Hendra Virus Infection

Hendra virus (HeV) belongs to a family of viruses called *Paramyxoviridae*, and are negative-sense, single-stranded RNA viruses in the order *Mononegavirales*. Given close homology and cross-reactivity on serological tests, HeV is commonly grouped with the Nipah Virus (NiV); together, these are referred to as *Henipavirus* (49). Hendra virus was discovered after an equine and human outbreak in Australia that resulted in the death of 13 horses and a horse trainer in 1994 (50). The very same year, another outbreak resulted in the death of a pair of horses and their owner in Australia who was admitted to the hospital with meningitis and was found on autopsy to have neuropathological evidence of CNS viral invasion (51). Pteropid fruit bats (flying foxes) are the primary reservoir host and infected horses act as intermediate hosts after exposure to bodily fluid, such as urine, feces or saliva, from an infected bat. Veterinarians and horse trainers are at particular risk for human infection thereafter.

Studies of acute human infection have demonstrated that a wide variety of tissues may be infected by HeV, including lung, liver, blood vessel, and brain (52). Humans infected with Hendra virus tend to first develop influenza-like and respiratory symptoms, which may be followed by systemic infection, encephalitis, and meningitis (53). Neuropathological findings have confirmed that HeV is neuronotropic and can cause meningitis or acute encephalitis which, in some cases, may be relapsing. Neuropathologic specimens from patients have revealed vasculitis, vasculitis-induced thrombosis, meningeal involvement, parenchymal inflammation, necrotic plaques and neuronal invasion (53, 54). Diagnosis of the systemic condition can be accomplished with serum ELISA or RT-PCR, although no tests specific to CNS infection have been studied (55).

Outbreaks have appeared to cluster in cooler months, a finding hypothesized to be related to the breeding cycles of flying foxes, or favorable survival of the virus in cooler and drier environments (56). In 2014, the Australian government approved a plan to destroy flying fox colonies in efforts to preempt further outbreaks. However, some have argued that disrupting colonies may increase the viral shedding among bats since increased stress may facilitate shedding in bat saliva (57, 58). Risk reduction strategies include an equine vaccine that became available in 2012; post-exposure treatment with neutralizing antibodies; and ecological interventions. Proposed ecological interventions include conservation/restoration of bat feeding habitats, to reduce risk of nutritional stress (which may potentiate salivary shedding) and urban colonization, and fencing horses away from trees at night to reduce exposures (56).

#### Influenza

*Influenza* are a group of RNA viruses of the *Orthomyxoviridae* family, which cause worldwide seasonal outbreaks of acute respiratory illness associated with significant morbidity and mortality. Seasonal cycles are occasionally dominated by pandemic strains of the virus, which cause devastating worldwide disease. There are four types of influenza viruses, A through D, which are characterized based on antigenic characteristics of viral surface proteins. Of these, types A and B primarily produce important human disease (59). The enduring infectivity of influenza comes from antigenic variation of its viral surface proteins, hemagglutinin and neuraminidase, which allow it to evade the human immune response to prior outbreaks. Influenza A is responsible for pandemic disease due to antigenic drift, in which viral surface proteins (particularly, hemagglutinin) are replaced with novel subtypes that are new to human hosts. This new genetic material often comes from waterfowl, an animal population that carry a large reservoir of influenza (60). In contrast, typical seasonal flu is a product of antigenic drift, which occurs in both influenza A and B, and refers to more subtle changes in surface proteins that come from accumulation of mutations, diminishing the antibody response elicited from prior outbreaks (60). Influenza B is carried primarily in human hosts, thus precluding major genomic changes. Though relatively rare, neurologic complications can result from both seasonal and pandemic outbreaks of influenza and compound the morbidity and mortality of the disease.

Neurological complications of influenza are widely reported in the pediatric population, but also occur in adults. Such complications can involve the CNS or PNS, and can range in severity from mild to severe, debilitating or fatal manifestations. Seizures are the most commonly reported neurologic complication, occurring in two-thirds of hospitalized pediatric patients who experience a neurologic complication. The majority of cases are febrile seizures, and in these cases the prognosis is generally good (61). Influenza-associated encephalopathy (IAE) is the second most common neurologic complication of influenza, though it is important to recognize that this term is non-specific and can present with non-focal signs such as changes in attention or arousal, as well as focal neurological findings. In most cases of IAE, there is no evidence of inflammation in the CSF and imaging studies are often unremarkable (61). RT-PCR has shown influenza virus in scattered cases, but the virus may be present in less than half of cases, suggesting a possible parainfectious mechanism of disease (62, 63). Determination of neurologic complications of influenza can thus be bolstered by demonstration of viral load in CSF, but its absence does not preclude the diagnosis.

Several IAE syndromes have been recognized and named based on specific clinical features, and in these cases, patients may be hospitalized specifically for neurologic care. Among the more specific and devastating of the IAE syndromes is acute necrotizing encephalopathy (ANE). ANE usually develops about 5 days after onset of typical viral symptoms, and is characterized by rapid deterioration in mental status, progressing to coma over the span of days. It is most strongly associated with influenza infection, although other viral infections have also been linked. Mild pleocytosis, elevated protein, and xanthochromia can be observed in the CSF (64, 65). Brain MRI characteristically shows bilateral necrosis of the thalami, although the cerebellum and brainstem can also be involved (61, 64). The prognosis is grim, and those who survive are often left with ongoing neurologic dysfunction, including motor deficits, intellectual disability, and epilepsy (64). Other important IAE syndromes reported include classical Reye's syndrome, which typically occurs in the convalescent phase of influenza and other viral illnesses, in children over 5 years of age. It is marked by rapidly progressive encephalopathy, associated with hepatic dysfunction. Affected children often develop coma and seizures. The underlying pathophysiology is thought to involve mitochondrial dysfunction (64). In some cases, the disease may be triggered by salicylate exposure. In the United States, prohibiting the use of salicylates in pediatric patients has been associated with a dramatic reduction in the incidence of Reye's syndrome (66). Imaging in Reye's syndrome is notable for diffuse cerebral edema, and laboratory tests will show evidence of liver dysfunction. CSF is characteristically non-inflammatory, thus, pleocytosis should argue against this diagnosis (61).

A spike in narcolepsy cases followed 1918 H1N1 influenza pandemic (67), and the same pattern appeared in northern Europe (68–71) and China (72) following the more recent 2009-10 H1N1 pandemic. The increase in incidence in northern Europe aligned with the pattern of Pandemrix influenza vaccine administration, which has been linked to immune-mediated narcolepsy (73, 74), although direct viral infection also has an independent effect as shown by the appearance of the syndrome after H1N1 infection in the absence of vaccine (68, 72, 75).

There are many more neurologic syndromes described following influenza infection, though the full list is beyond the scope of this article. Similar to the other presentations described, these syndromes are not singularly associated with influenza. They include acute disseminated encephalomyelitis (ADEM), transverse myelitis, acute inflammatory demyelinating polyneuropathy (AIDP) and the Miller-Fisher variant, as well as ischemic stroke. Of note, rare cases of AIDP and transverse myelitis have been associated with the influenza vaccine, though the relative risk is estimated to be much lower than the benefit of protection against active infection (61, 76).

One study of 34 children with acute necrotizing encephalopathy (including 11 influenza as prodrome) demonstrated a favorable outcome in 7 of 12 children with early steroid administration compared to 0 of 5 children without this treatment, although this was not statistically significant (77). Some case reports found benefit of steroids in pediatric influenza encephalopathy (78, 79) as well as adult influenza-associated acute necrotizing encephalitis (80). However, a larger retrospective cohort study of 692 children in Japan examined administration of corticosteroids early in the course of illness in children with influenza encephalopathy and did not show any benefit (81). The treatment of neurologic manifestations of influenza demands further research; the current evidence does not definitively support use of steroids (82).

#### Lassa Fever

Lassa fever is a hemorrhagic disease, caused by the single-stranded RNA Lassa virus hosted primarily by *Mastomys natalensis* rats and endemic to Western Africa. It initially infects immune cells in the nasopharynx, with subsequent spread to regional lymph nodes followed by multiorgan dissemination. After an incubation period of 1 to 3 weeks, its clinical course is heterogeneous; the majority of patients experience only malaise, headache, and low-grade fever, but more severe cases can include such diverse manifestations as diarrhea, hypotension, and pulmonary edema. Oozing blood from the oropharynx or, less commonly, the rectum or genitals, is seen in fewer than 20% of patients (83).

The most common neurologic complication of Lassa fever is sensorineural deafness, which has been reported in up to 25% to one-third of patients who survive the illness, although other research suggests this may be an overestimate (83, 84). Interestingly, the hearing loss usually occurs during the convalescent phase of the illness, sometimes after the patient is otherwise recovered, pointing to an immune cause of the disorder rather than a more direct viral cause (85). The hearing loss can be unilateral or bilateral and resolves spontaneously in fewer than half of cases. There appears to be no correlation between severity of illness and likelihood of hearing loss.

The less common CNS effects of Lassa fever are as heterogeneous as its systemic symptoms, including ataxia, meningitis, seizures, and coma (86, 87). The most common neuropsychiatric manifestation is depression but mania and psychosis are described (88).

Diagnosis of Lassa fever is commonly clinical in the context of appropriate signs and symptoms in an endemic region or following an appropriate exposure to a host animal. The disease can be diagnosed with high sensitivity and specificity with serum RT-PCR if logistically feasible, although the equipment required for this is expensive and requires significant operator training (89). Additionally, the high variation between lineages of the virus can make it poorly sensitive in some contexts (90).

In all cases of CNS involvement, symptomatic treatment of the disorders (i.e., antiepileptic drugs for seizures; antipsychotics for agitation in psychosis) appear to be the only indicated management. No proven treatment exists for amelioration of sensorineural deafness, and early treatment with ribavirin does not appear to protect against development of this symptom nor improve its chances of resolution.

### Marburg Virus Disease

Marburg virus forms its own genus within the family *Filoviridae* (91). It has a high mortality and is deemed by the U.S. Department of Homeland Security as a bioterrorism threat (92). The first recorded outbreak was in 1967 in Germany and Serbia. The most recent outbreak was in Uganda in 2017 (92). It is a hemorrhagic fever virus in the same family as Ebola virus, but it has only had three major outbreaks and a few reported cases to date (91). It is thought to be spread through mucosal transmission, breaks or abrasions of the skin, and parenterally, though the 1967 outbreak did isolate the virus in semen, and therefore, there is possibly sexual transmission (91). The virus has been isolated in blood, urine, and saliva (93).

The disease has three phases: a generalization phase, an early organ phase and a late organ or convalescence phase. The generalization phase consists of influenza-like symptoms of headache, chills, myalgias, and malaise, and the early organ phase consists of evidence of hemorrhagic disease. The late organ phase, which usually starts around day 13, is when most of the neurologic symptoms manifest, with reported convulsions, restlessness, obtundation, confusion, dementia, or coma (91). Patients in Germany were noted to have sullen, negative, and slightly aggressive behaviors (94).

Marburg virus may directly infect the CNS. Autopsies have shown glial nodule encephalitis in three of five cases in Germany (94). Neuropathology of hamster models have shown edema, enlargement of blood vessels in the brain, intracranial hemorrhage, and encephalitic lesions (91). RT-PCR can be used for diagnosis of the disease in principle, but no tests are commercially available and so, in practical terms, diagnosis is performed clinically (95).

#### Neisseria meningitidis

Meningococcal meningitis, caused by the gram-negative diplococci *Neisseria meningitidis*, is the most common cause of bacterial meningitis in young adults, and a major cause of both endemic and epidemic bacterial meningitis in children and adults worldwide (96). In the United States and other high-income countries, serogroups B, C, and Y are prevalent, while in the sub-Saharan African region known as “the meningitis belt,” repeated pandemics of serogroup A disease have occurred every 5 to 10 years (96). Vaccines available for the prevention of meningococcal infection include quadrivalent meningococcal polysaccharide conjugate vaccines for serogroups A, C, W, and Y (ACWY vaccine) and serogroup B conjugate vaccines. The capsular polysaccharide present in serogroup B resembles a protein found in human tissue, historically making vaccine development difficult. This has resulted in many individuals receiving quadrivalent ACWY vaccination while remaining vulnerable to serogroup B, although in the past 5 years the serogroup B vaccine has been increasingly effective and recommended (97–99).

The most common clinical presentations of meningococcal disease are meningitis and meningococcemia with sepsis. The classic features of meningococcal infection include a hemorrhagic rash, altered consciousness, and meningismus, however these may frequently not be present on a patient's initial presentation (100). Patients with meningitis generally have low serum concentration of meningococci with a high concentration in the CSF, whereas patients with meningococcal septicemia have the reverse findings, with high serum concentration of meningococci and endotoxin and low concentrations in the CSF (96).

Neurologic sequelae of meningococcal meningitis include focal neurologic deficits, hearing loss, seizures, cognitive impairment, vision loss, and hydrocephalus (101). Focal neurologic deficits occur less commonly in meningococcal meningitis compared to pneumococcal meningitis (3% of children and 2–9% of adults in meningococcal meningitis, 3–14% of children, and 11–36% of adults in pneumococcal meningitis) (101) and are most often secondary to cerebrovascular events (102). Hearing loss is similarly less common in meningococcal meningitis than in pneumococcal meningitis, and may be transient or permanent. Studies indicate a higher rate of hearing loss secondary to meningococcal meningitis in children in low-resource countries (19–23% of children) as compared to high-resource countries (about 4%) (101). Seizures are common in patients with bacterial meningitis, most often in the acute phase of illness, although some patients may have recurrent seizures after recovery. Patients recovering from meningococcal meningitis are at risk for long-term cognitive impairment, which can present as school challenges in children (103, 104) and cognitive slowing and memory impairment in adults (105), which may not improve.

The gold standard of diagnosis for *N. meningitidis* meningitis is CSF gram stain and culture, although the latter can be falsely negative as soon as 1–2 h after antibiotic administration. Several RT-PCRs have been developed that show high sensitivity and specificity even after antibiotic administration (106, 107), although these are rarely used in place of culture as they do not reveal antimicrobial sensitivities.

Treatment for meningococcal infection includes early antibiotic treatment, which results in rapid clearance of meningococci from the CSF (108). Aggressive management of complications such as shock or elevated intracranial pressure may also be required. Corticosteroids have only been shown to be effective in bacterial meningitis caused by *Streptococcus pneumoniae*, and so their use is appropriate early in the course of illness before pathogen speciation but are not indicated once *N. meningitidis* has been confirmed (109). With antibiotic treatment, the mortality rate for meningococcal meningitis has decreased to 7–10% (110, 111). Mortality is generally secondary to meningococcal septic shock or cerebral edema in the case of meningococcal meningitis (96).

#### MERS-CoV

Middle East respiratory syndrome-related coronavirus (MERS-CoV) is an enveloped, single-stranded, positive-sense RNA virus belonging to the diverse family *Coronaviridae*, which also includes SARS-CoV and 2019-nCoV. MERS-CoV was first identified as a human pathogen in 2012, when it was identified in a case of fatal pneumonia and renal failure in a 60-year old man in Saudi Arabia (112). Humans acquire the virus from dromedary camels, in which the virus is highly prevalent and can cause cold-like symptoms (113–115), although MERS-CoV has also been found in bats, a common coronavirus natural reservoir (116, 117). Molecularly, MERS-CoV uses the dipeptidyl peptidase 4 cell surface receptor protein, alternatively called DPP4 or CD26, to enter and infect cells (118, 119). As of November, 2019, 2,494 cases of MERS-CoV infection have been reported in humans, including outbreaks in 27 different countries, with an estimated case-fatality rate of 34.4% (120), making MERS-CoV the deadliest of the human coronaviruses. Clinically, infection can present variably; some people remain asymptomatic while others develop multi-organ involvement, with hallmark features including acute respiratory distress syndrome, renal failure, and coagulopathy. Risk factors for a more severe clinical course, as for other coronaviruses, include older age; presence of medical comorbidities such as hypertension, renal disease, or diabetes; and initial laboratory assessment consistent with a severe inflammatory response (121, 122).

Neurologic manifestations in MERS-CoV infection have been cited in small case series. In one examination of 70 MERS-CoV positive patients, 12.9% developed headache, 25.7% confusion, and 8.6% seizures (123). One series reported three cases of depressed arousal, vomiting, or focal motor deficits, with MRI abnormalities including patchy diffusion restriction and T2 hyperintensity in the subcortical and periventricular white matter, basal ganglia, thalami, midbrain, pons, cerebellum, and even extending into the cervical spinal cord (124). CSF analysis was performed in two of the three cases, and was notable for elevated protein, but normal glucose and cell counts. MERS-CoV RT-PCR in the CSF was negative. In a second series of four cases, one developed ophthalmoplegia and ataxia, without imaging, electromyogram, nerve conduction study or CSF abnormalities, including a negative MERS-CoV RT-PCR of the CSF (125). The three remaining cases in this series made note of presumed PNS involvement, with limb weakness, paresthesias, extremity pain, and hyporeflexia. Finally, two cases have been published noting neurological complications attributed to other organ system dysfunction in MERS-CoV, one of intracranial hemorrhage secondary to coagulopathy, and the second a length-dependent axonal polyneuropathy attributed to critical illness (126). Pathophysiology of the various neurologic manifestations of MERS-CoV, however, remains speculative.

#### Monkeypox

Monkeypox is a double-stranded DNA, zoonotic virus that characteristically causes a disease similar to smallpox, but with milder exanthem and lower mortality. The definitive reservoir has not been established, but it appears transmissible through monkeys as well as terrestrial rodents including rats and squirrels. Person-to-person transmission is possible but appears to occur at relatively low rates and result in a milder disease course (127, 128).

We have been able to find only two cases of neurologic complications of monkeypox in the literature. The individuals affected were both young girls who developed encephalitis, requiring intubation and mechanical ventilation. One 3-year-old girl died on day 2 of hospitalization while a 6-year-old girl survived after a 14-day course in the intensive care unit. The former patient had no CSF diagnostics performed, while the latter patient had detectable orthopoxvirus-reactive IgM in CSF (129, 130).

Of note, monkeypox appears to have a fatal course almost exclusively in infants and young children, specifically those who have not received vaccination against smallpox (129).

#### Nipah Virus Infection

Nipah virus (NiV) is an enveloped, negative-sense single-stranded RNA *Paramyxovirus* of the genus *Henipavirus*, endemic to Southeast Asia and Africa (131, 132). First isolated around 1999 after a series of outbreaks in Malaysia and Singapore, it owes its name to the Sungai Nipah village in Malaysia (133). Bats of the genus *Pteropus* act as its primary reservoir, and NiV seropositivity has been found among *Pteropus* populations as widely distributed as West Africa, Madagascar, and across southeast Asia (132, 134, 135). NiV from asymptomatic bats can cause human infection either by direct exposure to bat secretions on fruit/sap or via a wide range of symptomatic intermediate host animals (pigs, horses, dogs, cats) (136).

NiV infection presents primarily with a fulminant encephalitis (55, 132, 137). After a 5–21 day incubation period, a patient typically presents with high fever, headache and meningismus (132, 138). Although rare during the initial Malaysian outbreak, an accompanying viral pneumonia has been noted in more than half of cases during recent outbreaks in Bangladesh and India (139). After neurologic symptom onset, mental status may decline rapidly, often progressing in just 24–48 h to severe encephalopathy and coma; convulsive seizures are common, and mortality is high (132). Outbreak case fatality rates have varied widely between 40 and 90%, notably with lower rates with initial Malaysian outbreaks and higher rates with recent outbreaks in Bangladesh and India (138, 140, 141). Infection is typically monophasic, but reports describe cases of relapsing fulminant encephalitis years after the initial infection in some patients, even those whose initial symptoms were mild (142). CSF chemistry profile generally resembles other non-hemorrhagic viral meningitides. MRI shows diffuse and patchy, often punctate, subcortical and deep white matter lesions, less often with cortical and brainstem involvement, typically with lesions less than 1 cm in diameter (143). Pathology demonstrates a necrotizing small-vessel-predominant vasculitis present both diffusely in the CNS as well as in the heart, lung, kidney, and most other organs sampled (144). Diagnosis can be performed with RT-PCR or ELISA; while the gold standard is detection of neutralizing antibodies against NiV in serum, the virus is classified as a level 4 biosafety agent and so this is not generally viable in a clinical setting (55, 145). Treatment is largely supportive, as antiviral trials to date have shown equivocal or no therapeutic effects (146–148).

The first recognized outbreak is thought to have occurred on Malaysian pig farms, where asymptomatic bats in tree branches hanging above farms transmitted NiV in their saliva and other secretions to pigs below leading to rampant pig-pig and pig-to-human transmission via respiratory and other secretions (149). More than 300 human cases and 105 patient deaths occurred (150). With the exception of one nurse with no direct pig contact demonstrating asymptomatic NiV seropositivity and MRI brain findings typical for infected persons (151, 152), evidence of human-to-human transmission during initial outbreaks was extremely limited.

Since this, outbreaks have been recognized in Bangladesh and India nearly annually since 2001 from a related and potentially more dangerous strain of NiV, with additional outbreaks in the Philippines stemming from NiV-infected horses (132, 149, 153, 154). Human case clustering in these recent NiV outbreaks supports a concerning new feature of person-to-person transmission (132, 155–157). In some recent outbreaks, such as the 2018 Kerala, India outbreak during which 16 of 18 confirmed cases died, the overwhelming majority of cases and fatalities were due to human-to-human transmission after one or a few index animal-human transmissions (155, 158, 159). These developments raise concern for an increased risk of large-scale and lethal pandemic spread, prompting designation of NiV by the WHO to a high-risk “priority disease” (160, 161).

No NiV-protective vaccines are currently commercially available, although host animal and human Nipah virus vaccines are currently in development. Recent small-scale preclinical studies with these vaccines have reported effective immunity after VZV-platform vaccination of non-human primates (131, 162, 163). Ribavirin treatment was associated with a significantly lowered risk of mortality in one cohort study of 140 individuals in Malaysia (146), although a smaller study in Kerala showed no difference (164). Until and potentially even after any future success in widespread vaccination, the Nipah Virus will likely continue to threaten human populations for the foreseeable future given the stable, geographically widespread and genetically diverse range of animal (bat) reservoirs, which themselves serve important ecological roles, as well as the wide array of susceptible intermediate hosts including pigs, horses, dogs, and cats (132, 140).

#### Novel Coronavirus (2019-nCoV, SARS-CoV-2)

In December 2019, a series of viral pneumonia cases of unknown cause appeared in Wuhan, Wubei, China, and deep sequencing analysis from lower respiratory tract samples indicated that the infection was due to 2019 novel coronavirus (2019-nCoV) (165). The 2019-nCoV (COVID-19) is a betacoronavirus in the same subgenus as the severe acute respiratory syndrome (SARS) virus (see below), and therefore, also referred to as SARS-CoV-2. The structure of COVID-19's receptor-binding gene region is similar to that of the SARS coronavirus, and COVID-19 uses the same angiotensin-converting enzyme 2 (ACE2) receptor for cell entry (166).

It is too early to tell if there is increased prevalence of neurological complications as a result of COVID-19 when compared to other respiratory viruses. Among a subset of patients with COVID-19 in three hospitals in Wuhan, China: 36% of patients had neurologic manifestations with CNS manifestations in 25%, PNS manifestations in 9%, and skeletal muscle injury in 11% (167). Multiple retrospective case series in hospitalized patients report CNS manifestations that include dizziness, headache, and encephalopathy (168). Additionally, smell and taste disturbances are characteristic of COVID-19, leading some to speculate CNS entry via the olfactory mucosa as a possible route of central nervous system entry (167, 169). Given the severity of pulmonary disease seen in some patients with COVID-19, there is also a theory as to whether the brainstem could be affected through infection of the nucleus of the solitary fascicle, which modulates respiratory effort in response to atmospheric carbon dioxide and oxygen concentration, although there has been no concrete evidence of this to date (170).

Some case series and case reports have described peripheral nerve and muscle complications of COVID-19 that can be seen in other viral infections. GBS was reported in both Wuhan (171) and Italy, without identification of COVID-19 in the CSF, and of note, not all patients had elevated cells in the CSF (172). A case of acute myelitis in COVID-19 infection in Wuhan is reported, but no CSF profile or spine imaging are included (173). A case of COVID-19-associated myopathy caused by type I interferonopathy has been reported (174). Rhabdomyolysis has also been reported as an atypical presentation of COVID-19 (175): 20% of patients with severe respiratory disease and 5% with non-severe respiratory illness had evidence of skeletal muscle injury in a retrospective cohort of COVID-19 patients (167).

Diagnosis of systemic COVID-19 is most commonly performed with RT-PCR on nasopharyngeal swabs, serology tests, and antibody-based rapid tests (176, 177). Some case reports and case series have reported detection of SARS-CoV-2 via RT-PCR in CSF of COVID-19-positive individuals with neurologic symptoms (178–180), although this finding has also been absent in many individuals who have severe COVID-19 infection with neurological symptoms (181–183). Furthermore, a reanalysis using the Xpert®–Xpress SARS-CoV-2 test of stored CSF samples performed in three individuals who initially had virus detected in CSF by RT-PCR showed no subsequent evidence of virus (184). It thus remains to be seen what role measurement of virus in CSF plays in clinical management of individuals with COVID-19.

We anticipate studies focused on the impact of SARS-CoV-2 infection on neurological function and the long-term neurological sequelae will continue to be published and our understanding will deepen over the coming years. At this point, it is obvious that extrapulmonary manifestation of SARS-CoV-2 includes neurological symptoms and disease, and will be crucial for clinicians to recognize and treat.

#### Plague

*Yersinia pestis* is a zoonotic, gram-negative, non-motile organism transmitted by the tropical rat flea (*Xenopsylla cheopis*). The plague disease it causes has three clinical subtypes: bubonic, septicemic, and pneumonic. The most common bubonic form is characterized by sudden onset of malaise, dizziness, high fever, and lymphadenitis. This can progress to septicemic plague, which includes a heterogeneous phenotype of hypotension plus other systemic manifestations; approximately 80–90% of septicemic plague cases occur with preceding buboes (enlarged lymph nodes) (185). The most commonly reported neurological complication of plague is meningitis, which appears to arise in the context of delayed treatment of primary bubonic or septicemic plague.

The pathogenesis of plague meningitis is presumed due to hematogenous dissemination from buboes, and in light of this some studies have proposed that axillary buboes predispose to meningitis given easier access to lymphatic tracts leading to intracranial compartments (186). The case series published to date have not found a statistically significant effect for this. Development of septicemic plague has also been cited as a risk factor for meningitis, but similarly no statistically significant findings have been published.

The first documented case of CNS involvement of plague was in 1940, when a 3-year-old with no known immunosuppression developed headache, cough, and fever over 6 days, subsequently developing signs of meningismus and with microscopic evaluation of CSF consistent with *Y. pestis* (187). Subsequent reports have also included a significant number of pediatric patients. One case series detailed a 7-year-old girl who presented with malaise and buboes, and subsequently developed encephalopathy and meningismus followed by death; as well as a 47 year-old woman who presented with malaise and fever, then progressed to develop a waxing-waning delirium and Kernig's sign in the setting of fever (188). A retrospective analysis examined a cohort of 105 patients from 1970 to 1979 in whom 6 patients (6%) developed neurologic complications; 5 of those 6 patients were children aged 10 to 15 years old, while the remaining patient was a 35 year-old woman (189). A case series of 27 plague patients in Arizona documented 3 individuals (11%) with meningitis, all between 2 and 8 years of age (190).

In most cases studied, clinical signs and symptoms of meningitis appeared after onset of systemic symptoms, with a delay ranging from a few days to two or more weeks. Diagnosis is generally with *Y. pestis* culture from bubo aspirate, sputum, or blood, depending on the type of plague with which an individual presents, although rapid antigen-based tests are also available (185). Since the majority of case reports examining plague meningitis are from the 1970s or earlier, imaging features of neurologic disease are not well characterized.

#### Rift Valley Fever

Rift Valley Fever Virus (RVFV) is a negative-sense, single-stranded RNA virus that belongs to the genus *Phlebovirus* (191). RVFV is primarily transmitted to livestock through arthropods, primarily the *Aedes aegypti* mosquito. While humans can be infected by mosquito bite, the primary route of infection is due to direct or indirect contact with infected animal blood and products, possibly through aerosolization (192).

Rift Valley Fever (RVF) was first identified in East Africa in 1931 with nine recognized outbreaks between 1999 and 2010 (193) and a spreading geographic distribution that extends from Madagascar to Saudi Arabia. RVFV was identified as a potential biologic weapon by the USA prior to termination of a biologic weapons program in 1969 (194). There have been no cases of human-to-human transmission (195).

In humans, most cases of RVF are mild and can cause a non-specific flu-like illness with fever, muscle pain, joint pain and headache. However, severe cases include ocular, meningoencephalitis or hemorrhagic fever forms. Neurologic manifestations are identified in up to 17% of patients in some outbreaks, which can manifest with encephalopathy, ataxia, vertigo, seizures, and coma (196). Retinitis can occur in one or both eyes with the affected eye showing macular edema and exudates. Some cases demonstrate vasculitis, retinal hemorrhage or infarcts (192). Meningoencephalitis has a delayed onset of presentation (2–60 days) with fever, headache, cranial nerve deficits, seizures, hemiparesis, visual hallucinations, choreiform movements, and locked-in syndrome all reported (192, 197). Histopathology of brain lesions shows focal necrosis with infiltration of lymphocytes and macrophages with perivascular cuffing (192). A case report described MRI brain findings of multiple bilateral asymmetrical cortical hyperintense areas as well as CSF characterized by elevated protein and lymphocytic pleiocytosis (198). RVFV has not been isolated in human CSF, but has been found in animal studies (199).

Diagnosis for RFV is through RT-PCR of viral RNA or ELISA for IgM antibodies against RVF virus (200). Treatment for RVF is mainly supportive. Ribavirin was tried during outbreaks. Newer antivirals are being tested on animal models. A live attenuated vaccine exists primarily for veterinary use. A human inactivated vaccine was trialed for high risk exposures, but is not widely approved for commercial use (191).

#### SARS

Severe acute respiratory distress syndrome (SARS) is a viral respiratory illness caused by a type of coronavirus called SARS-associated coronavirus (SARS-CoV) (201). Coronaviruses are enveloped non-segmented positive-sense RNA viruses belonging to the family *Coronaviridae* (165). Infections are usually mild but two betacoronaviruses, SARS-CoV and MERS-CoV (see above) are associated with higher mortality rates, with SARS-CoV, estimated by the WHO, to have had a global case fatality of around 9.6% (202). SARS was the first major novel infectious disease to affect countries internationally in the twenty-first century, and is reported to originate from southern China in 2002 (203).

There has been concern that SARS may translocate and infect the CNS, with reports of isolation of the virus in the brain (204) and specifically in neurons from autopsies of eight confirmed SARS patients (205). Isolation of the virus through RT-PCR of the CSF was performed in a case of a woman with SARS who experienced convulsions (203). An additional case of RT-PCR detection occurred when a woman with SARS presented with status epilepticus; of note, in this case, the CSF showed no white cells and normal protein, i.e., a non-inflammatory picture (206). SARS has also been isolated in the CSF of a child with ADEM. There are reports of increased rates of depression, anxiety, and posttraumatic symptoms after SARS infection (207).

In murine models, SARS has been correlated to a possible chronic demyelinating condition that resembles multiple sclerosis (208). Murine models have also shown SARS enters the brain primarily through the olfactory bulb. The infection results in transneuronal spread to the brain (209).

The most sensitive modality for SARS diagnosis is ELISA for IgM against the virus, although this can remain negative in infected patients for weeks into illness (210). RT-PCR has been tested for patients earlier in the course of disease, although sensitivity on many of these tests may be as low as 50% (211, 212).

Similar to COVID-19, there are neuromuscular complications in patients with SARS, including skeletal muscle injury and rhabdomyolysis, although these manifestations are theorized to be related to a cytokine storm rather than direct infection by the virus (213).

#### Smallpox

Smallpox is caused by the variola virus, which is a double-stranded DNA virus in the *Orthopoxvirus* genus. Although it was eradicated in 1980, there is concern that smallpox could reemerge as a bioterrorism agent. Human to human transmission predominantly occurs through airborne droplets, but can also occur through infected vesicles, scabs, and fomites. It is highly contagious. Transmission in a household can be up to 60% (214). Diagnosis is with serum RT-PCR (215).

Smallpox can incubate for 1–2 weeks and patients are most infectious within 10 days of developing lesions (216). The most typical manifestation of smallpox, termed *Variola major*, starts with a prodrome of fever, malaise, headache, and delirium before development of skin lesions. Lesions occur after 24 h, initially on mucous membranes (enanthem) followed by a cutaneous rash (exanthem) starting in the face and spreading to the extremities that becomes vesicular then pustular. Mortality is estimated at 30–50% (217). *Variola minor*, cause by a similar strain of Variola virus, leads to milder disease with estimated 1% mortality (217, 218).

Encephalopathy is common in clinical presentations of smallpox (216). Acute perivenular demyelination was found in patients who have died of smallpox (217, 219). Encephalitis can develop around 6–10 days after presentation and occur in ~1 in 500 patients with variola major and ~1 in every 2,000 patients with variola minor (219, 220). Patients can also report severe headaches, hallucinations and delirium. Smallpox has also been associated with ocular complications, most often binocular, which can lead to blindness (217). CSF initially shows a lymphocytic or neutrophilic pleocytosis, which becomes a lymphocytic pleocytosis with moderately elevated protein and normal glucose (219).

Post-vaccination encephalitis is a rare but severe complication (221). In patients less than 2 years of age, encephalitis incidence is estimated at 0 to 103 in 100,000 person-years and occurs 6–10 days after infection. In patients over 2 years old, the incidence is estimated at 2 to 1,219 per 100,000 person-years and occurs 11–15 days after infection (217). Mortality from post-vaccine encephalitis can be as high as 25% (222). Other neurologic complications associated with the vaccine include headache, seizures, cranial nerve palsy, GBS, hemiplegia, and coma (222). Pathology shows hyperemia, lymphocytic infiltration of the meninges, and degeneration of the basal ganglia (217).

#### Tularemia

The *Francisella* genus is a group of gram negative bacilli, of which *F. tularensis* is a highly virulent subtype responsible for the majority of the human disease known as tularemia. It is a fastidiously growing bacterium, which was first isolated after an outbreak of a rodent-borne illness that occurred in 1911 in Tulare County, California following the San Francisco Earthquake. During the outbreak, the organism was grown from cultures obtained from infected ground squirrels. *F. tularensis* is found almost exclusively in North America; disease outside North America occurs in the Northern Hemisphere, and is attributed to the *holarctica* subspecies (223). Though human disease is rare, *F. tularensis* is recognized to be among the most infectious of pathogens, with a dose of only 10 organisms capable of causing infection with a high mortality rate when untreated. Given its potential lethality and ease for dissemination through airborne delivery, the Centers for Disease Control and Prevention (CDC) has recognized *F. tularensis* as a potential bioweapon (224).

Transmission largely occurs through two possible disease cycles: terrestrial or aquatic. Terrestrial cycles rely on rabbits and hares as hosts, with arthropods such as ticks and flies as vectors. Aquatic life cycles rely on mammals, such as beavers and muskrats, who shed the live organism into bodies of water, where it can infect exposed humans (223). The disease can also be directly transmitted from close contact with host mammals, and multiple mechanisms have been recognized: the processing and consuming of contaminated meat, hamster bites, and lawn care (running over infectious material from an animal, or the animal itself with a lawn mower or weed-whacker can disseminate the organism through aerosolization) are a few reported scenarios (223). In Martha's Vineyard, Massachusetts, an outbreak of tularemia was observed during the summer of 2000, prompting case-control studies of prior cases that showed lawn mowing and brush cutting as risk factors (225). Importantly, healthcare and laboratory workers who process infected specimens are also at risk for disease (223).

Tularemia infection can vary in presentation based on host factors, bacterial virulence, and how the disease was transmitted. The incubation period can vary considerably, though typically lasts 3 to 6 days. Clinical manifestations are most simply categorized as ulceroglandular or typhoidal, though pneumonic presentations are increasingly common and can occur with both types. Ulceroglandular disease is the more common form, composing about 75% of cases, and has several overlapping subtypes. Classically, it is characterized by a skin lesion associated with regional, tender lymphadenopathy. History often reveals recent contact with possible host mammals or arthropod vectors through insect bites. The skin lesion is thought to represent the site of inoculation, and can appear as an eschar with surrounding erythema (226). Exposure to the eye and pharynx can cause localized manifestations with conjunctivitis or pharyngitis, which may be accompanied by lymphadenopathy. Typhoidal tularemia is less common and is characterized by high fever with hepatosplenomegaly. There is usually an absence of skin or lymph node involvement, although often there is associated gastrointestinal and pulmonary disease (223). Diagnosis is through antibody titers in serum, with a 4-fold increase between acute and convalescent values representing a positive result. Specific titer cutoffs in the acute phase of illness can also be used to diagnose the disease even without matching serum samples from convalescence (227).

Given the overall rarity of tularemia infection, its neurologic complications are largely the subject of case reports. Both classic and Miller Fisher variants of GBS are described (228, 229), though some cases are disputed to represent a manifestation of infectious polyneuritis, critical illness polyneuropathy, or myopathy (230). Meningitis is more commonly reported. It is rarely complicated by cerebral abscesses (231). A 61-year-old patient with gait difficulties and ataxia on examination was found to have tularemia and was diagnosed with cerebellitis. His neurologic difficulties completely improved with treatment of infection (232). Ventriculoperitoneal shunt (VPS) infection due to tularemia has been identified in a child with medical history of myelomeningocele requiring VPS who later disclosed that, 2 weeks prior to becoming ill, he had helped his friends skin a rabbit (233).

All clinicians must maintain a high index of clinical suspicion for tularemia, as serologic testing depends on the identification of antibodies against the organism, which are typically not detectable until at least 2 weeks after the onset of illness. Gram stain and culture rarely detect the organism (234).

#### Yellow Fever

Yellow fever virus (YFV) is a positive-sense, single-stranded RNA virus that belongs in the *Flavivirus* genus. It is predominantly transmitted by mosquitoes and is endemic to tropical regions of Sub-Saharan Africa and South America. While an effective live-attenuated vaccine has been developed that protects against YFV, there continue to be occasional outbreaks predominantly in Africa, South America, and Asia where immunization levels are low (235).

YFV incubates for 3–6 days after transmission and can lead to a mild non-specific febrile illness. However, a portion of patients (~14%) can develop severe disease leading to hemorrhagic fever and multi-organ failure (236). Diagnosis can be performed with either RT-PCR or ELISA of serum, depending on when during the time course of infection the testing takes place (237, 238). Neurologic manifestations are rare, but early studies described delirium, seizures, and coma. Elevated intracranial pressure can also occur with CSF demonstrating elevated protein and absence of inflammatory cells. The brain tissue does not show severe inflammatory changes, but can show petechiae, perivascular hemorrhages, and edema (239). As a result, the CNS changes were thought to be due to cerebral edema or metabolic factors rather than direct inflammation (236, 239). Neuroinvasion leading to encephalitis appears to be exceedingly rare with only case reports of optic neuritis, cranial nerve palsy, and paralysis (236). However, YFV has been detected in human CSF (240). A later case series examining severe yellow fever cases admitted to the ICU found that seizures occurred in 24% of patients without evidence of significant cerebral edema on CT imaging (241). There is a paucity of literature describing neurologic manifestations of yellow fever, especially given an effective vaccine.

Severe neurologic manifestations have been associated with the yellow fever vaccine. While rare, neurotropic disease associated with the vaccine 17D is estimated at 0.8 per 100,000 people vaccinated, especially in individuals >60 years old (242). Neurotropic disease manifestations range from headache and encephalopathy to encephalitis, demyelinating disease, and GBS (243).

#### Zika Virus Disease

Zika Virus (ZIKV) is a positive-sense RNA flavivirus first isolated and identified in Uganda in 1947 (244). ZIKV is widely distributed in warm climates with separate African and Asian lineages, though few cases in humans were reported before the spread of the Asian lineage occurred in Yap in 2007, French Polynesia in 2013, and the Americas in 2015 (245). The ZIKV virus is primarily transmitted by *Aedes* mosquitoes (246), although it can also be spread by sexual contact (247), blood transfusion, or maternal-fetal transmission (246). Symptomatic ZIKV infection occurs in 20–50% of infected patients and usually consists of a non-specific rash, low-grade fever, arthralgia, myalgia, and conjunctivitis (248). Diagnosis is generally with RT-PCR followed by ELISA for IgM in patients with equivocal results or those with negative results and sufficient clinical suspicion (249, 250). ZIKV gained significant attention during the American pandemic from 2015 to 2017 due to rapid spread of the American subclade of the Asian lineage throughout a susceptible population and the recognition of severe neurological complications.

Neurologic symptoms of ZIKV infection in adults can be divided into peripheral and central manifestations. Peripherally, ZIKV infection is associated with GBS, which was estimated to affect 2–3 patients per 10,000 ZIKV infections (251). Symptoms of GBS began 5–10 days after ZIKV illness. Acute inflammatory demyelinating polyneuropathy, acute motor sensory axonal neuropathy, Bickerstaff's encephalitis, and the Miller-Fisher syndrome subtypes of GBS have all been reported after infection (252). CNS ZIKV disease is less common compared to ZIKV-associated GBS and may manifest as an encephalomyelitis (253) or vasculitis leading to strokes.

Fetuses of women infected with ZIKV during pregnancy are at risk of congenital Zika syndrome, and ZIKV-associated microcephaly. Four to seven percentage of infections will result in fetal loss. Twenty to thirty percentage of the fetuses of women infected with ZIKV will develop infection (254). Infected fetuses are at risk for congenital ZIKV syndrome, which consists of a disruption in cortical development and subsequent collapse of the skull, resulting in microcephaly. Affected infants may also have subcortical calcifications, chorio-retinal atrophy, pigmented mottling of the retina, arthrogryposis, and hearing loss (255).

## Discussion

Diseases with the potential for epidemic and pandemic spread have a variety of neurological manifestations, ranging from rare presentations involving the CNS to fulminant manifestations that define the disease outbreak. While some diseases represent regional epidemics, several reflect pandemic diseases. The role of the neurologist and neurologically-focused clinician is critical for early identification of outbreaks and characterization of the range of features of systemic disease-causing pathogens.

Each disease listed includes CNS involvement although in some cases the situation is atypical (e.g., plague) or the data reflect few cases in vulnerable groups (e.g., cholera). Six of the diseases (Chikungunya, COVID-19, *F. tularensis*, influenza, MERS, and Zika virus) additionally include a recognized element of PNS symptom burden. The most common CNS manifestation is a non-specific encephalopathy, although a number of diseases cause more syndromic neurologic features that are pivotal in the disease diagnosis such as in the fulminant encephalitis of Nipah virus, the ophthalmoplegia and ataxia of MERS-CoV-2, and the ICH of Crimean-Congo hemorrhagic fever or Marburg virus disease. The most common PNS manifestation of the diseases on this list is post-infectious AIDP.

As climate change continues to disrupt host and vector habitats, diseases with regional epidemic potential have risk of reaching new populations and locations. One example of this is Crimean-Congo hemorrhagic fever but others are also recognized (26, 27). It is thus crucial that healthcare providers globally are able to recognize the clinical manifestations of these diseases so that appropriate treatment can be initiated promptly at an individual level while pathogen spread is attenuated at a population level.

The range of evidence for the pathogens is variable and tends to focus on case reports and case series. While two diseases (monkeypox, tularemia) are studied in only a handful of individual case reports, all other pathogens include at least moderately sized case series and often large, population-scale reviews indicating a significant burden of neurologic disease.

The mechanism of neurological involvement is variable among the diseases. Twelve of the associated pathogens have potential evidence of direct neurotropism as shown either through identification in CSF during active infection or neuropathologic findings (*V. cholerae*, ebolavirus, influenza virus, Hendra virus, Lassa virus, *N. meningitidis*, Nipah virus, *Y. pestis*, SARS, *F. tularensis*, yellow fever virus, Zika virus). Four pathogens have possible neurotropism as shown either through presence in the brains of animal models but not humans (Chikungunya virus, Crimean-Congo hemorrhagic fever virus, Rift Valley fever virus) or by presence of antibodies but not the pathogen itself in CSF of affected individuals (Marburg virus). In four pathogens, the question of direct neurotropism remains unclear (monkeypox, MERS, smallpox, SARS-CoV-2). Many of the diseases show evidence of parainfectious neurologic syndromes in addition to any direct neurotropism they may have (Chikungunya, influenza, Lassa fever, COVID-19, smallpox, tularemia, Zika).

Of the 20 diseases on the list, seven (Chikungunya, COVID-19, influenza, MERS, Neisseria meningitis, plague, tularemia, and Zika virus), have spread to all or nearly all WHO world regions, often despite having well-identified origins in particular locations. Vaccines are presently available for only eight of the diseases (cholera, COVID-19, ebola virus disease, influenza, Neisseria meningitis, plague, smallpox, yellow fever), while two others have a vaccine available for animal hosts (Hendra virus) or under development for humans (rift valley fever virus) (Table 1). Of the remaining 10 diseases, none have curative treatment and many have potentially fatal outcomes, highlighting the importance of control of disease spread following recognition.

Our work has several limitations. We provide a scoping review but not a systematic review. Our summary is limited by the review being performed of English language articles with searches performed through PubMed and related medical search engines online. We did not include historical literature, for example on plague or smallpox, that may be earlier than our search was able to extend. By the nature of this topic, the data reviewed came from a variety of regions with differing availability of neurological expertise, testing techniques, disease surveillance, and access to healthcare which led to a lack of standardization of some results. Although we attempted to be exhaustive in reporting the range of infections, due to the broad scope of our task and high number of manuscripts available on some pathogens, we highlight the most critical information for neurologists in the field.

Future efforts at prevention of the spread of these pathogens—and the epidemics and pandemics that they are capable of causing—should span investments in science, education, clinical care, and public health. Directions for the medical community could include increased funding for vaccine research as well as strengthening medical facilities with the ability to address and evaluate disease surges where and when they arise. Pragmatic efforts include public education on the best methods to prevent the spread of disease including face masks, hand hygiene, quarantine, and proper handling of biospecimens, burials, and autopsies. Effective and unified population-level messaging could decrease morbidity and mortality of the individual patient and prevent further spread while reducing the burden on healthcare systems (256–261).

While the diseases on the WHO list have the potential to cause widespread harm, many of them fortunately have distinct clinical and diagnostic features that make it possible to identify outbreaks when they are suspected, and early interventions by providers can significantly impact the trajectory of a pathogen's spread. This review has outlined the neurological features of all of the currently listed diseases with epidemic and pandemic potential by the WHO with the goal of assisting clinicians who see neurological conditions with the prompt identification of diseases. This list may evolve and expand over time as new pathogens are discovered, pathogenicity changes, or hosts and conditions for spread continue to change. One lesson that is crucial to glean from the high morbidity and mortality of the COVID-19 pandemic is that the interconnectedness of the modern world makes the threat of any of these 20 diseases real and imminent, and the medical community must be vigilant in their surveillance to ensure they do not spread.

## Author Contributions

FM was additionally involved with project supervision. All authors contributed to the manuscript conception, drafting, and editing for critical content.

## Conflict of Interest

The authors declare that the research was conducted in the absence of any commercial or financial relationships that could be construed as a potential conflict of interest.
